# Female Medical Students

**Published:** 1887-11

**Authors:** 


### Female Medical Students.

A humorous piece of versification, recently published in a contemporary, describes the pitiable condition of a north country "coach" on being applied to by a female student to give her private lessons in anatomy. The gist of the humor resides in the bashfulness of the grinder, who was eminently desirous of restricting his lessons to things which in his estimation were "consistent wi' propriety." As a joke, the sentiment is commendable, but when it is brought into real life it becomes ridiculous. Men who have studied abroad can bear witness to the fact that it is at least as easy to study anatomy, or any other department of medical education, in the society of female students, with the most perfect propriety, as it is to perform gynæcological or obstetrical operations in an operating theatre with the assistance of the nurses, without any mental qualms. So long as separate courses of study are insisted upon for the two sexes, so long necessarily will a certain amount of diffidence mark the manner of both when they come together in after life. We are, however, ready to confess that so far as our experience goes, the diffidence has generally been more marked on the part of the male than on that of the female student, and this fact is explained by the latter having schooled themselves to ignore everything not having a scientific bearing. Female medical students do not belong to that category of young ladies, one of whom is reported to have fainted on being told that a man was looking at her with a naked eye.—*Med. Press and Circular*, July 20, 1887.

